# Investigation of the Protective Effects of Magnesium on Bupivacaine-Induced Toxicity at the Level of Colon Cell Culture

**DOI:** 10.3390/biomedicines12081652

**Published:** 2024-07-24

**Authors:** Ceren Önal, Kemal Tolga Saraçoğlu, Ayten Saraçoğlu, Beyza Nur Özkan, Eray Metin Güler, Gülten Arslan, Seçil Azime Karakuş, Yekbun Bulun, Tomasz Gaszynski, Pawel Ratajczyk

**Affiliations:** 1 Department of Anesthesiology and Reanimation, Ağrı Research and Training Hospital, Ağrı 04200, Türkiye; 2 Department of Anaesthesiology, ICU & Perioperative Medicine, Hazm Mebaireek General Hospital HMC, Qatar University College of Medicine, Doha P.O. Box 2713, Qatar; kemaltolgasaracoglu@gmail.com; 3 Department of Anaesthesiology, ICU & Perioperative Medicine, Aisha Bint Hamad Al Attiyah Hospital HMC, Qatar University College of Medicine, Doha P.O. Box 2713, Qatar; saracogluayten@gmail.com; 4 Department of Medical Biochemistry, Hamidiye Faculty of Medicine, University of Health Sciences Türkiye, İstanbul 34480, Türkiye; beyzanur156@gmail.com (B.N.Ö.); eraymetinguler@gmail.com (E.M.G.); 5 Department of Anesthesiology and Reanimation, University of Health Sciences Türkiye, Kartal Dr. Lütfi Kırdar City Hospital, İstanbul 34865, Türkiye; gulten.arslan@yahoo.com.tr (G.A.); secil.usul@hotmail.com (S.A.K.); 6 Department of Anesthesiology and Reanimation, Bingöl State Hospital, Bingöl 12000, Türkiye; dr.ybulunn@hotmail.com; 7 Department of Anesthesiology and Intensive Therapy, Medical University of Lodz, 90-419 Łódź, Poland; pawel.ratajczyk@umed.lodz.pl

**Keywords:** cytotoxicity, DNA damage, apoptosis, bupivacaine, magnesium

## Abstract

The primary objective of this in vitro study was to prevent the risk of toxicity associated with bupivacaine, widely used in clinical practice, by using magnesium (Mg), a readily available and cost-effective element, as an adjuvant. We hypothesized that Mg might exhibit a protective effect against cytotoxicity in a colon cell culture model under conditions of bupivacaine-induced LAST. Our secondary aim was to investigate its effect on genotoxicity, apoptosis, and iROS. CCD-18Co cells were used in our study. Control group (group C), Bupivacaine group (group B), Magnesium group (group M), and Bupivacaine+Mg group (group BM) were created. The viability of CCD-18Co cells incubated for 24 h in group C was determined to be 100%. These cells were evenly divided, and bupivacaine was administered to group B at concentrations of 5 to 300 μM. In group M, doses of Mg at 0.625 to 320 mEq were added. It was determined that the maximum viability was observed at a Mg dose of 40 mEq (*p* < 0.05). In group BM, bupivacaine was administered at the same concentrations in combination with Mg (40 mEq), and cell viability was measured. DNA damage, apoptosis, and iROS were assessed at concentrations of bupivacaine by administering 40 mEq Mg. In group B, viability decreased dose-dependently in CCD-18Co (*p* < 0.05, *p* < 0.01, *p* < 0.001). In group BM, the viability decreased in cells at increasing concentrations compared to group C (*p* < 0.05, *p* < 0.01, *p* < 0.001), but the viability was affected positively compared to group B (*p* < 0.05). In group B, DNA damage increased (*p* < 0.05, *p* < 0.001). In group BM, DNA damage increased (*p* < 0.05, *p* < 0.001). However, in group BM, DNA damage levels were reduced compared to group B (*p* < 0.05, *p* < 0.01). In group B, apoptosis increased (*p* < 0.05, *p* < 0.001); in group BM, apoptosis increased (*p* < 0.001) compared to group C. However, in group BM, apoptosis decreased compared to group B (*p*< 0.05). iROS increased in group B (*p* < 0.05, *p* < 0.01, *p* < 0.01) and group BM (*p* < 0.05, *p* < 0.01, *p* < 0.001) compared to the group C. However, in group BM, iROS decreased in comparison to group B (*p* < 0.05). In conclusion, Mg exhibits a protective effect against bupivacaine-induced toxicity.

## 1. Introduction

With the wider adoption of ultrasound in our clinical practice, the popularity of regional anesthesia procedures has increased. However, during operations rising in number, complications ranging from localized nerve damage to systemic cardiovascular toxicity and even death may occur. Local Anesthetic Systemic Toxicity (LAST) is a potentially fatal complication that can occur with all local anesthetics regardless of the route of administration. LAST incidence during peripheral nerve blocks varies between studies, ranging from 0.04% to 0.18% [1]. The American Society for Regional Anesthesia and Pain Medicine (ASRA) recommends using lipid emulsions in cases of LAST with a risk of developing cardiac arrest [2]. It has been reported that while lipid emulsions are effective, further research is needed to determine the optimal timing during toxicity [3]. Considering patient safety, the prevention of LAST takes precedence over its treatment.

Bupivacaine can cause serious systemic toxicity, especially cardiotoxicity, through the induction of arrhythmias, poor cardiac contractility, and/or even cardiac arrest, which is difficult to reverse [4,5]. Bupivacaine can decouple mitochondrial oxidative phosphorylation and inhibit respiratory chain complexes I, III, and IV in the mitochondrial oxidative respiratory chain, thus enhancing reactive oxygen species (ROS) production [6]. Recent reports demonstrate that bupivacaine disrupts targets of classical insulin signaling, including protein kinase B (Akt) and ribosomal protein s6 kinase 1, in cellular models [7,8,9]. Beyond Akt, bupivacaine activates other controllers of glucose homeostasis, including 5′ adenosine monophosphate-activated protein kinase (AMPK) [10,11]. It has been reported in cell culture studies that toxicity leads to apoptosis by increasing the levels of reactive oxygen species (ROS) and intracellular calcium, as well as causing mitochondrial membrane and DNA damage [12,13,14,15].

Magnesium (Mg) plays a crucial role in the mechanisms underlying vitality and is essential for homeostasis. Mg is involved in practically every major metabolic and biochemical process within the cell and is responsible for numerous functions in the body, including bone development, neuromuscular function, signaling pathways, energy storage and transfer, glucose, lipid and protein metabolism, DNA and RNA stability, and cell proliferation [16]. Due to its crucial role in regulating cellular time processes and sustaining circadian rhythm, Mg has recently been recommended in the treatment protocols for inflammatory bowel disease [17]. Mg sulfate possesses analgesic properties as it can inhibit calcium influx into cells and antagonize N-methyl-d-aspartate (NMDA) receptors in the central nervous system. Therefore, it is commonly used as an adjuvant to local anesthetic agents in central and peripheral blocks [18].

The primary objective of this study was to prevent the risk of systemic toxicity associated with bupivacaine, widely used in clinical practice, by using Mg, a readily available and cost-effective element, as an adjuvant. We hypothesized that Mg might exhibit a protective effect against cytotoxicity in a colon cell culture model under conditions of bupivacaine-induced LAST.

## 2. Materials and Methods

This study protocol was approved by the Institutional Review Board (IRB), University of Health Sciences Turkey Hamidiye Scientific Research Ethics Committee on 2 July 2021, with approval number 23/4.

### 2.1. Materials

Human colon fibroblast cell lines (CCD-18Co, CRL-1459™) were obtained from the American Type Culture Collection (ATCC^®^, Manassas, VA, USA). The Cell Titer-Glo^®^ viability kit was purchased from Promega Corporation (Madison, WI, USA) and particulate bupivacaine (73360-54-0, Sigma-Aldrich, Istanbul, Türkiye). Low melting agarose, normal melting agarose, Mg, ethidium bromide, and acridine orange were acquired for the experiment from Sigma (St. Louis, MO, USA). Trypsin-EDTA, fetal bovine serum (FBS), penicillin-streptomycin, and Eagle’s Minimum Essential Medium (EMEM) were procured from Gibco (Gibco Co., Billings, MT, USA). All utilized chemicals were provided in a form suitable for cell culture, ensuring compatibility with sterile laboratory conditions.

### 2.2. In Vitro Studies

#### 2.2.1. Maintenance of Cells

Human colon fibroblast (CCD-18Co) cell lines were cultured in EMEM complete medium at 37 °C with 5% CO_2_ conditions. The completed medium was prepared sterile, containing 89% EMEM, 10% FBS, and 1% penicillin/streptomycin. Cell passage was conducted using 0.25% trypsin EDTA, and cell counting was performed utilizing a Thoma slide with trypan blue. Some of the cells that went to the upper passages were cryopreserved and stored in a liquid nitrogen tank. The cells were incubated at 37 °C for 24 h to investigate the cytotoxic, genotoxic, and apoptotic effects of bupivacaine and Mg applied to the cells seeded on the plates in the experimental stages. All experiments were performed in quadruplicate.

#### 2.2.2. Study Design

In the study, four different groups were created based on the hypothesis that Mg may have a protective effect against the cytotoxic, genotoxic, and apoptotic effects of bupivacaine. The groups are as follows:

Control Group (Group C): As a control group, CCD-18Co cells were given 1xdPBS.

Bupivacaine Group (Group B): Bupivacaine alone was applied to CCD-18Co cells. The experimental dosages of bupivacaine were established at ten discrete levels spanning the range of 5 to 300 μM (5, 10, 20, 40, 60, 80, 100, 150, 200 and 300 μM).

Mg Group (Group M): In this group, CCD-18Co cells were given Mg at ten different doses (0.625, 1.25, 2.5, 5, 10, 20, 40, 80, 160, and 320 milliequivalents (mEq)).

Additionally, the half-maximal effective concentration (EC_50_) was calculated for Mg after cell viability experiments. This dose was used in combination with bupivacaine in the experiments. To assess its protective efficacy, the Mg concentration of 40 mEq was selected, and cell viability experiments were conducted on the bupivacaine + Mg combination (group BM).

Bupivacaine + Mg Group (Group BM): Cells treated with bupivacaine in the range of 5 to 300 μM concentrations and receiving the EC_50_ dose of Mg (40 mEq) constitute this group.

### 2.3. Measurements

#### 2.3.1. Cell Viability

This study employed a luminometric ATP test to detect cytotoxicity in CCD-18Co cells. The commercially purchased CellTiterGlo^®^ (Promega Corporation, Madison, WI, USA) luminescence cell viability kit is a homogeneous method based on the amount of ATP, indicating the presence of viable cells. The principle of the method is that since the amount of ATP is proportional to the number of viable cells, luciferin in the environment converts to oxyluciferin by the recombinant luciferase enzyme in the presence of ATP in the cells, emitting luminescence. CCD-18Co cells were seeded at a density of 15 × 10^3^ cells/well in a white opaque 96-wellplate and left in the incubator for 24 h at 37 °C with 5% CO_2_ to obtain their morphology. Then, the cells were incubated with bupivacaine concentrations (group B) ranging from 5 to 300 Μm or Mg concentrations (group M) ranging from 0.625–320 mEq for 24 h. No treatment was applied to the cells in group C, and the cells in this group were used as a control. After incubation, ATP solution was added, and within 5 min, luminescence values were measured using a luminometric method on a multi-plate reader (Synergy HTX Multi-Mode Reader with Gen 5 1.11 software, Biotek, Winooski, VT, USA). Luminescence detected in the presence of ATP was measured in relative luminescence units (RLU) with reference to group C, where cell viability was considered 100%. The EC_50_ values were determined for Mg through nonlinear regression analysis of response curves. The doses in groups were repeated four times consecutively.

#### 2.3.2. Measurement of Intracellular ROS

Intracellular ROS (iROS) production was assessed using the fluorescent signal indicator 2′,7′-dichlorodihydrofluorescein diacetate (H2DCF-DA). The colorless H2DCF-DA is oxidized by iROS in the environment and converted into green fluorescent dichlorofluorescein (DCF). There is a correlation between the increasing amount of iROS and the emitted fluorescence. In group BM, 96-well black opaque plates were seeded with a density of 15 × 10^3^ cells/well. After 24 h, bupivacaine was added at concentrations of 5–300 mEq, along with Mg at a dose of 40 mEq, which provided maximum proliferation. After incubation, the medium was aspirated and washed three times with 1x phosphate-buffered saline (PBS) and then treated with 100 µL of 10 µM H2DCF-DA prepared in ddH_2_O, followed by an incubation at 37 °C for 30 min. The fluorescence intensity of the resulting DCF after incubation was measured using a fluorescence plate reader (Varioskan Flash Multimode Reader, Thermo, Waltham, MA, USA) with excitation/emission wavelengths set at 488 nm/525 nm. The results were calculated relative to ATP by comparing them to group C with 0.1% DMSO added (iROS/ATP), and four replicates were performed for each dose.

#### 2.3.3. Measurement of DNA Damage

Genotoxic damage, specifically DNA damage, was measured using the single-cell gel electrophoresis method, also known as the Comet Assay, developed by Singh et al. [19]. The Comet Assay method relies on the principle that DNA migrates differently in an electric field based on its varying electrical charge and weight. In this method, cells are embedded in agarose and subjected to lysis. If there is no damage (genotoxicity) in the DNA, the appearance of the DNA is compact, and no tail (comet) is formed. However, if DNA has undergone damage and fragmentation, these resulting fragments will have different electrical charges and molecular weights. Therefore, when stained with the fluorescent dye ethidium bromide, these DNA fragments will exhibit diverse mobility in the electrophoretic medium, creating a comet-like appearance [19,20]. To determine the genotoxic activity of bupivacaine and bupivacaine with Mg, cells were seeded in 6-well plates in group B and group BM at a density of 50 × 10^3^ cells/well. Concentrations of bupivacaine (5–300 μM) and the same concentrations in combination with the maximum proliferative dose of Mg (40 mEq) were applied and incubated for 24 h. After 24 h, the cells were removed with trypsin-EDTA. The cells, washed with 1xPBS, were centrifuged at 500 ×*g* for 5 min at +4 °C, and the supernatant was discarded. Ten microlitersof cell suspension was mixed with 85 µL of 0.65% low melting agarose (LMA). The mixture was then added to slides pre-coated with 1% normal melting agarose (NMA). After freezing, the samples were incubated in a lysis solution at +4 °C for 4 h. After incubation, the samples were washed with cold 1xPBS and incubated at +4 °C in electrophoresis buffer for 40 min to facilitate the opening of DNA strands. Subsequently, the samples were electrophoresed at +4 °C, with a voltage of 25 V and a current of 250 mA, for 20 min. After the slides were washed three times in neutralization buffer, they were fixed with ethanol. Ethidium bromide (2 μg/mL) was then applied to the dried samples, and images were captured using a fluorescence microscope (Nikon ECLIPSE Ts2, Nikon, Tokyo, Japan). The DNA tail percentages in the images were analyzed using the Comet Assay IV analysis program. Results are expressed as % tail intensity.

#### 2.3.4. Measurement of Apoptosis

Acridine orange/ethidium bromide (AO/EB) staining is a dual staining method utilized for the assessment of morphological changes in cells. Acridine orange is a vital dye that stains both live and dead cells. On the other hand, ethidium bromide stains only cells that have lost membrane integrity. Healthy cells exhibit a homogeneous green color. Apoptotic cells containing both AO and EB appear orange in color. However, necrotic cells have a different nuclear morphology and dye density as they contain condensed chromatin, unlike healthy cells, and appear to be distorted red [21]. Cells were seeded in 6-well plates in group BM at a density of 50 × 10^3^ cells/well. Concentrations of bupivacaine (5–300 μM) and the same concentrations in combination with the EC_50_ dose of Mg (40 mEq) were applied and incubated for 24 h. After 24 h, the cells were removed with trypsin-EDTA. The cells, washed with 1xPBS, were centrifuged at 500 ×*g* for 5 min at +4 °C, and the supernatant was discarded. Ten microliters of cell pellet and 10 µL of AO/EB solution (100 µg/mL AO + 100 µg/mL EB) were added to an empty slide at a 1:1 ratio, and a coverslip was added. Images were evaluated and recorded under a fluorescence microscope (Nikon ECLIPSE Ts2, Nikon, Tokyo, Japan). A minimum of 100 cells were counted at each concentration in randomly picked cells. Apoptotic cells are given as % apoptosis.

### 2.4. Power Analysis

The required sample size for the study was determined through a power analysis. Based on previous studies used as references, with a 95% confidence level and 80% power (considering a mean difference of 4.97 units and a standard deviation of 1.8 units), a minimum of four replicates were performed for each dose. For the 96-well plates, a minimum of 15 × 10^3^ cells were seeded per well, and for the 6-well plates, a minimum of 50 × 10^3^ cells were seeded per well.

### 2.5. Statistical Analysis

All statistical analyses were conducted using the SPSS software package for Windows (Version 25, Chicago, IL, USA). Results are presented as mean ± standard deviation. Data from all experiments were analyzed for statistical significance using analysis of variance (One-Way ANOVA). The EC_50_ values for Mg on cell lines were calculated using non-linear regression analysis. The associations between all parameters were analyzed using Pearson’s correlation coefficient. A *p*-value of < 0.05 was considered statistically significant.

## 3. Results

In group B, the impact of applied bupivacaine concentrations on the viability of healthy colon cells in this in vitro culture study is presented in Table 1. It was found that bupivacaine concentrations significantly reduced viability in healthy cells. The viability started to significantly decrease for healthy colon cells at 10 μM bupivacaine concentrations (*p* < 0.05). The decrease in cell viability was correlated with the increasing concentrations of bupivacaine. (*p* < 0.05 for 10–20 μM, *p* < 0.01 for 40–60 μM, *p* < 0.001 for 80–300 μM).

In group M, the impact of Mg on the viability of healthy colon cells is presented in Table 2. The statistical analysis revealed that increased Mg doses initially led to an elevation in viability in healthy colon cells, followed by a significant decrease. The viability for CCD18-Co started to show a statistically significant increase at a dose of 20 mEq Mg (*p* < 0.05). The increase in cell viability showed a correlation up to a dose of 40 mEq Mg. Cell viability exhibited maximum proliferation at a dose of 40 mEq Mg, which was statistically significant for CCD18-Co (*p* < 0.05). Cell viability showed a statistically significant decrease at Mg doses of 80 mEq and above for CCD18-Co (*p* < 0.01 for 80 mEq, *p* < 0.001 for 160 mEq and 320 mEq).

In group BM, the impact of bupivacaine concentrations and the combination with the maximum proliferative Mg dose (40 mEq) on the viability of healthy colon cells is presented in Table 3. This combination significantly reduced viability at increasing doses for healthy colon cells. A statistically significant decrease in cellular viability started for CCD18-Co at 20 μM bupivacaine concentrations (*p* < 0.05). A statistically significant and severe decrease in cellular viability was observed for combinations of the maximum proliferative Mg dose (40 mEq) with 40–300 μM bupivacaine concentrations for CCD18-Co compared to group C (*p* < 0.01 for 40 and 60 μMp < 0.001 for 80–300 μM). Statistical analysis of cell viability revealed a significant difference between group B and group BM. Cell viability in group BM appeared to be increased compared to group B at concentrations between 10 and 60 μM (*p* < 0.05). When administered in combination with bupivacaine, Mg has a protective effect up to a concentration of 60 µM but has a toxic effect at concentrations higher than 60 µM.

**Figure 1:**
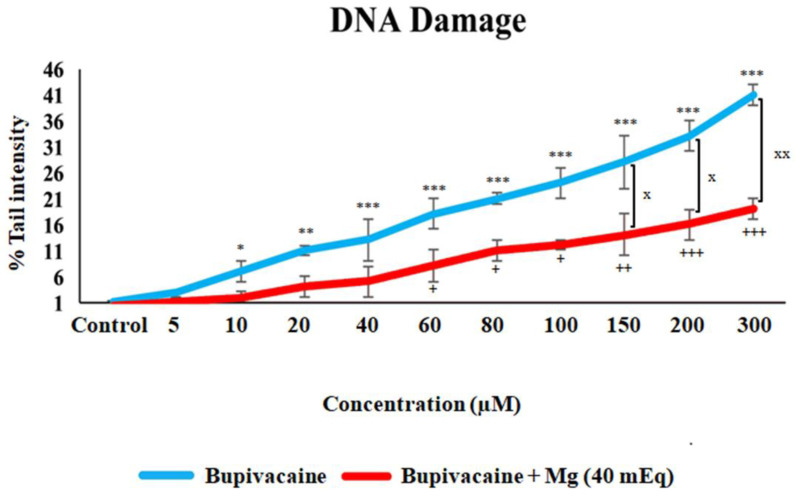
The effect of bupivacaine and bupivacaine in combination with magnesium (40 mEq) on DNA damage levels in healthy colon cells (Mg: magnesium). *p* < 0.05 was considered statistically significant. * *p* < 0.05, ** *p* < 0.01, *** *p* < 0.001; + *p* < 0.05, ++ *p* < 0.01, +++ *p* < 0.001; ^x^ *p* < 0.05, ^xx^ *p* < 0.01.

**Figure 2:**
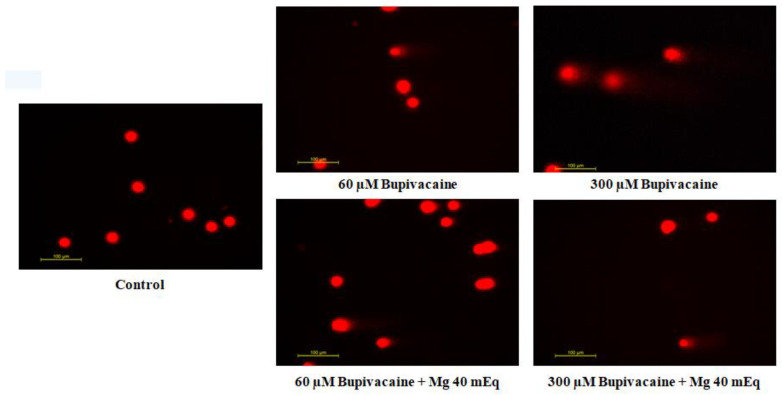
Fluorescence microscope images of bupivacaine and bupivacaine in combination with magnesium (40 mEq) on DNA damage levels in healthy colon cells (Mg: magnesium).

The effect of bupivacaine and combinations of bupivacaine with Mg (40 mEq) on the level of DNA damage in healthy colon cells (CCD18-Co) is presented in Figure 1 and Figure 2. DNA damage levels in CCD18-Co cells significantly increased at 10 and 20 μM bupivacaine concentrations (*p* < 0.05 * for 10 μM, *p* < 0.01 * for 20 μM) and severely increased at 40 μM to 300 μM bupivacaine concentrations (*p* < 0.001 ***) compared to group C. DNA damage levels significantly increased in group BM compared to group C (*p* < 0.05 ^+^ for 60–100 μM, *p* < 0.01 ^+^ for 150 μM, *p* < 0.001 ^+^ for 300 μM). However, there is statistical significance between group B and group BM. In group BM, DNA damage levels reduced at concentrations of 150–300 μM compared to group B (*p* < 0.05 ^x^ for 150 and 200 μM, *p* < 0.01 ^xx^ for 300 μM).

**Figure 3:**
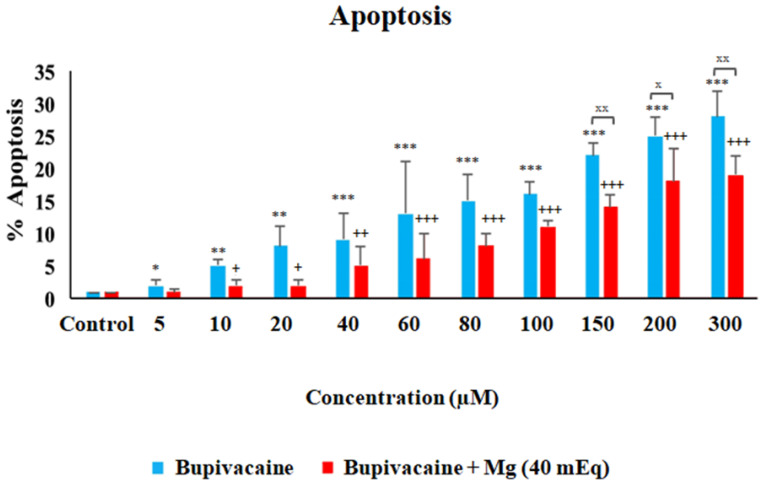
The The effect of bupivacaine and bupivacaine in combination with magnesium (40 mEq) on apoptosis levels in healthy colon cells (Mg: magnesium). *p* < 0.05 was considered statistically significant. * *p* < 0.05, ** *p* < 0.01, *** *p* < 0.001; ^+^ *p* < 0.05, ^++^ *p* < 0.01, ^+++^ *p* < 0.001; and ^x^ *p* < 0.05, ^xx^ *p* < 0.01.

**Figure 4:**
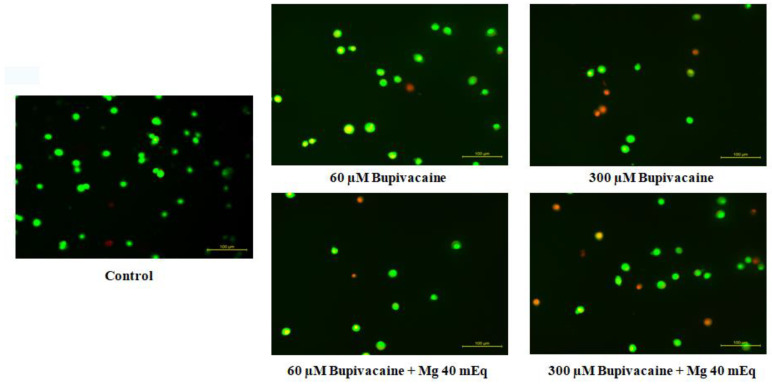
Fluorescence microscope images of bupivacaine and bupivacaine in combination with magnesium(40 mEq) on apoptosis levels in healthy colon cells (Mg: magnesium).

The effect of bupivacaine and combinations of bupivacaine with Mg (40 mEq) on apoptosis in healthy colon cells (CCD18-Co) is presented in Figure 3 and Figure 4. Apoptosis in CCD18-Co cells was significantly elevated in group B compared to group C (*p* < 0.05 * for 5 μM, *p* < 0.01 ** for 10 and 20 μM, *p* < 0.001 *** for 40–300 μM). In group BM, apoptosis significantly increased compared to group C (*p* < 0.05 ^+^ for 10 and 20 μM, *p* < 0.01 ^++^ for 40 μM, *p* < 0.001 ^+++^ for 60–300 μM). However, considerable statistical significance was observed between group B and group BM at concentrations of 150–300 μM. In group BM, apoptosis decreased compared to group B. (*p* < 0.05 ^x^ for 200 μM, *p* < 0.01 ^xx^ for 150 and 300 μM).

**Figure 5:**
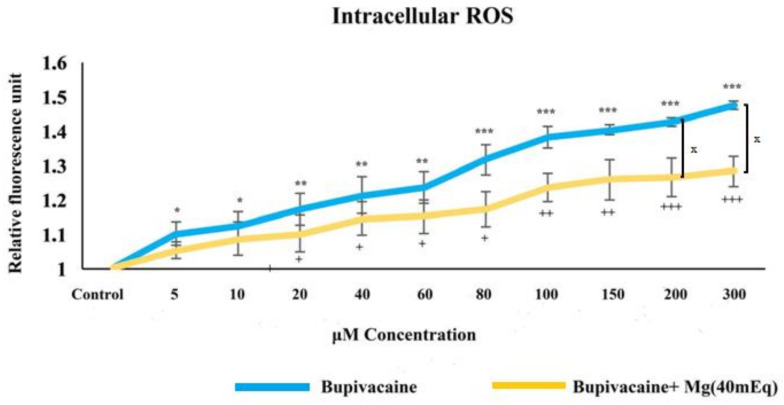
The effect of bupivacaine and bupivacaine in combination with magnesium (40 mEq) on intracellular ROS levels in healthy colon cells (Mg: magnesium). *p* < 0.05 was considered statistically significant. * *p* < 0.05, ** *p* < 0.01, *** *p* < 0.001; ^+^ *p* < 0.05, ^++^ *p* < 0.01, ^+++^ *p* < 0.001; and ^x^ *p* < 0.05.

The effect of bupivacaine and combinations of bupivacaine with Mg (40 mEq) on iROS levels in healthy colon cells (CCD18-Co) is presented in Figure 5. In group B, there was a significant increase in iROS levels in CCD18-Co cells compared to group C (*p* < 0.05 * for 5–10 μM, *p* < 0.01 * for 20–60 μM, *p* < 0.001 * for 80–300 μM). In group BM, iROS levels exhibited a significant increase compared to group C (*p* < 0.05 ^+^ for 20–80 μM, *p* < 0.01 ^+^ for 100 and 150 μM, *p* < 0.001 ^+^ for 200 and 300 μM). Cell viability in group BM appears to be increased compared to group B at concentrations between 10 and 60 μM (*p* < 0.05). When administered in combination with bupivacaine, Mg has a protective effect up to a concentration of 60 µM but has a toxic effect at concentrations higher than 60 µM.

## 4. Discussion

In this study, induction of in vitro tissue toxicity by the local anesthetic was accomplished through the application of increasing bupivacaine concentrations to cell culture. Cytotoxicity was observed in healthy colon cells. Evidence of DNA damage and apoptosis were recorded. An optimal Mg concentration was determined to be effective in reversing these toxicity findings. Using Mg as an adjuvant at the optimal dose was shown to effectively enhance cell viability in the systemic toxicity of local anesthetics.

In previous in vitro studies, bupivacaine has been demonstrated to reduce viability. A study reported a dose-dependent decrease in viability in rabbit annulus fibrosus and nucleus pulposus cells with the application of bupivacaine [22]. In another study, a reduction in viability was shown in bovine chondrocyte cells at a bupivacaine concentration of 0.5% [23]. The positive effect of magnesium on viability has been observed in studies. In a study, applying a neurotoxin called MPP+ (1-methyl-4-phenylpyridinium) resulted in an initial increase in magnesium, and blocking the passage of Mg to these cells led to a decrease in cell viability [24]. In another study, when Mg was applied in vitro to neural stem cells, proliferation increased by 38% [25]. In the same study, these cells were transplanted into in vivo models, and proliferation and viability were observed to increase by 103% in the in vivo environment. In a different study involving human chondrocyte cells, cells were initially treated with levobupivacaine and bupivacaine at a concentration of 0.5% and ropivacaine at a concentration of 0.75%, and then Mg was added. It was observed that the addition of Mg increased viability in a dose-dependent manner. However, it was demonstrated that increasing doses of Mg have the opposite effect, inducing toxicity [26]. Another in vitro study conducted on human choriocarcinoma cells reported a protective effect of Mg against lipopolysaccharide-induced toxicity, enhancing viability within a narrow therapeutic range [27].

The apoptotic cell count in cells has been found to increase with both higher bupivacaine concentrations and prolonged exposure. In an in vitro study on human breast cancer and healthy epithelial cells, bupivacaine was reported to effectively increase apoptosis in both groups, with a higher impact observed in cancer cells [14]. It was demonstrated in a study conducted on cancerous colon cells that bupivacaine did not affect apoptosis at clinical doses but increased apoptosis at higher doses [28]. Another study on cancerous colon and skin cells showed that bupivacaine increased apoptosis in cancerous colon cells but did not significantly affect cancerous skin cells [29]. However, no study is currently available regarding bupivacaine’s impact on apoptosis in healthy colon cells. Our study indicated that increasing concentrations of bupivacaine applied to healthy colon cells would increase apoptosis, parallel to the effect observed in cancer cells in previous studies. While inducing apoptosis in cancer cells has a negative impact on cancer progression, resulting in positive outcomes for the organism, an increase in apoptosis in healthy cells may lead to an increase in cell death. However, it is clear that there is a need for further research to explore the potential negative consequences of this. We also highlighted the apoptosis-reducing effect of Mg. In the study by Cappadone et al., an indole derivative was introduced into human colon cells. When Mg was administered to these cells after cytotoxic and apoptotic effects were created with the agent, it was found that apoptosis decreased via cytochrome c in mitochondria [30]. Kızıldağ et al., in their research on healthy rats, oral Mg was given to the rats for 8 weeks, and its effect on the hippocampus was examined. They found that the long-term effect of Mg induced apoptosis by increasing the Bax/Bcl-2 ratio [31]. As shown in the aforementioned studies, Mg has a significant effect on apoptosis. While its effect is generally anti-apoptotic, as we have shown, some studies have shown that it has the opposite effect and increases apoptosis. The effect of Mg is still open to debate and indicates the need for further research.

In rat dorsal root ganglion cells exposed to a high-glucose environment, the administration of bupivacaine has been observed to elevate ROS levels and trigger autophagy [15]. In another study on rabbit intervertebral disc cells, the application of bupivacaine led to an increase in intracellular ROS levels. The administration of N-acetyl-L-cysteine, a ROS inhibitor, reduced intracellular ROS and subsequently blocked cell death [32]. Magnesium has been shown to enhance cell viability in HT22 mouse hippocampal cells treated with amyloid-beta peptide (Aβ25-35), which induces neurotoxic effects. Additionally, it was observed that magnesium reduced ROS levels, thereby inhibiting oxidative stress [33]. In an in vivo study involving uremic rats with chronic kidney failure, it was observed that rats fed a diet containing 0.1% Mg experienced increased pro-inflammatory cytokines like TNF-α, IL-1β, and IL-6, resulting in the induction of oxidative stress.

Conversely, when the same rats were fed a diet containing 0.6% Mg, there was a reduction in the pro-inflammatory response and oxidative stress [34]. In our investigation, it was observed that elevated bupivacaine concentrations led to an increase in intracellular ROS levels. Notably, we identified that an optimal Mg dose of 40 mEq could reverse these effects. This value was considered a threshold, and even with increasing bupivacaine concentrations, the combination treatment significantly reduced ROS levels.

## 5. Conclusions

Bupivacaine induced cytotoxicity and triggered DNA damage and apoptosis in colon cells. As a result, cell viability decreased. Mg, when administered in combination with bupivacaine at the dose of 40 mEq, the dose that maximizes proliferation, has been found to significantly increase cytotoxicity, genotoxicity, and apoptosis compared to the control group. But in comparison to bupivacaine alone, it has been found that the combination of bupivacaine and Mg has reduced cytotoxicity, genotoxicity, and apoptosis. In conclusion, Mg exhibits a protective effect against bupivacaine-induced toxicity.

## Figures and Tables

**Table 1:** Impact of bupivacaine on viability in healthy colon cells.

Bupivacaine	CCD-18Co	p
Concentration	(Avg. ± SD) ^a^	
(μM)		
Control	100 ± 0	*p* > 0.05
5	96.5 ± 4.65	*p* > 0.05
10	94.63 ± 8.76	*p* < 0.05
20	90.84 ± 8.79	*p* < 0.05
40	85.97 ± 8.89	*p* < 0.01
60	81.10 ± 8.91	*p* < 0.01
80	78.23 ± 11.07	*p* < 0.001
100	69.33 ± 3.70	*p* < 0.001
150	63.45 ± 2.77	*p* < 0.001
200	59.57 ± 7.12	*p* < 0.001
300	42.68 ± 9.35	*p* < 0.001

Abbreviations: CCD-18Co: healthy colon cell, %: percentage, Avg.: average, SD: standard deviation. ^a^ Data are presented as average ± standard deviation.

**Table 2:** Impact of magnesium on viability in healthy colon cells.

Magnesium Dosage	CCD-18Co	*p*
(mEq)	(Avg. ± SD) ^a^	
Control	100 ± 0	*p* > 0.05
0.625	101 ± 14.47	*p* > 0.05
1.25	102 ± 11.29	*p* > 0.05
2.5	105 ± 9.32	*p* > 0.05
5	109 ± 8.76	*p* > 0.05
10	113 ± 8.86	*p* > 0.05
20	117 ± 7.69	*p* < 0.05
40	120 ± 9.93	*p* < 0.05
80	108 ± 5.27	*p* < 0.01
160	89 ± 4.67	*p* < 0.001
320	50 ± 5.15	*p* < 0.001

Abbreviations: CCD-18Co: healthy colon cell, %: percentage, Avg.: average, SD: standard deviation. ^a^ Data are presented as average ± standard deviation.

**Table 3:** Impact of the combination of bupivacaine and magnesium (40 mEq) on the viability of healthy colon cells.

Bupivacaine Concentration (μM) + Magnesium (40 mEq)	CCD-18Co (Avg. ± SD) ^a^	*p*
Control	100 ± 0	*p* > 0.05
5	99 ± 13.17	*p* > 0.05
10	97 ± 11.00	*p* > 0.05
20	91 ± 13.41	*p* < 0.05
40	85 ± 5.71	*p* < 0.01
60	81 ± 11.56	*p* < 0.01
80	77 ± 4.16	*p* < 0.001
100	60 ± 8.23	*p* < 0.001
150	53 ± 9.01	*p* < 0.001
200	44 ± 2.46	*p* < 0.001
300	41 ± 5.03	*p* < 0.001

Abbreviations: CCD-18Co: healthy colon cell, %: percentage, Avg.: average, SD: standard deviation. ^a^ Data are presented as average ± standard deviation.

## Data Availability

The data that support the findings of this study are available from the corresponding author upon reasonable request.
